# Explaining One-Dimensional Convolutional Models in Human Activity Recognition and Biometric Identification Tasks

**DOI:** 10.3390/s22155644

**Published:** 2022-07-28

**Authors:** Gustavo Aquino, Marly G. F. Costa, Cicero F. F. Costa Filho

**Affiliations:** R&D Center in Electronic and Information Technology, Federal University of Amazonas, Manaus 69077-000, Brazil; gustavoaqui@gmail.com (G.A.); mcosta@ufam.edu.br (M.G.F.C.)

**Keywords:** human activity recognition, grad-cam, convolutional neural networks, biometric user identification, deep learning, accelerometer data, explainable AI

## Abstract

Due to wearables’ popularity, human activity recognition (HAR) plays a significant role in people’s routines. Many deep learning (DL) approaches have studied HAR to classify human activities. Previous studies employ two HAR validation approaches: subject-dependent (SD) and subject-independent (SI). Using accelerometer data, this paper shows how to generate visual explanations about the trained models’ decision making on both HAR and biometric user identification (BUI) tasks and the correlation between them. We adapted gradient-weighted class activation mapping (grad-CAM) to one-dimensional convolutional neural networks (CNN) architectures to produce visual explanations of HAR and BUI models. Our proposed networks achieved 0.978 and 0.755 accuracy, employing both SD and SI. The proposed BUI network achieved 0.937 average accuracy. We demonstrate that HAR’s high performance with SD comes not only from physical activity learning but also from learning an individual’s signature, as in BUI models. Our experiments show that CNN focuses on larger signal sections in BUI, while HAR focuses on smaller signal segments. We also use the grad-CAM technique to identify database bias problems, such as signal discontinuities. Combining explainable techniques with deep learning can help models design, avoid results overestimation, find bias problems, and improve generalization capability.

## 1. Introduction

Smartphones and wearable devices provide unprecedented opportunities to monitor human physiological signals, creating possible applications in different areas such as activity tracking, ambient assisted living, healthcare, and others [1]. The global wearable-device market is projected to grow at 11.3% by 2025, resulting in a revenue of $62.82 million [2]. In recent decades, several papers have been published, with different techniques and solutions in HAR, using the wide variety of sensors incorporated into a smartphone, such as accelerometers, gyroscopes, microphones, GPS units, and others. Mobile devices use these sensors to register data and apply personal assistants to monitor and help the users in their exercise and activity routines, making them healthier. For example, it is known that simple physical activities such as sitting, walking, running, climbing, or descending stairs can help reduce the risk of chronic diseases such as obesity, diabetes, and cardiovascular diseases [3]. With HAR techniques, it is possible to create an assistant that can monitor a user’s daily activities and improve his or her health conditions by making recommendations based on the detected activities and encouraging the user to exercise more. 

Another use of HAR is in ambient assisted living. As the population’s average age worldwide has increased considerably, healthcare needs of the elderly, such as physical rehabilitation, physical support, and home care [4], have been increasing. In this context, many studies have been done on fall detection [5,6,7]. These systems can monitor older people and help decrease their medical expenses, increase their independence, and improve their quality of life.

HAR can be implemented using a variety of sensors. We can use inertial measurement units (IMU), cameras, microphones, and GPS, among others. Although studies use cameras in HAR, this is not a predominant approach due to user privacy concerns. Among wearable devices, smartphones are the most widely used [4]. Smartphones are affordable and widespread. Furthermore, they are already integrated into the daily routine, making them even more interesting for HAR. With them, no additional device is needed. The smartphone is one of the first devices people come in contact with when they wake up [8]. Developing a smartphone app is more accessible than other wearables, such as smartwatches. There are many different open-source repositories to use as a base, either for collecting data from onboard sensors, running and inferring machine learning models locally, or sending the data to a cloud architecture. In addition, the smartphone typically has more processing power than other devices, which helps it to collect data efficiently and execute deep learning models.

The IMU is the most widely used in HAR among the available sensors. The IMU is an electronic device that can report position variations and provide time series data. It is typically an integrated sensor package that includes an accelerometer, gyroscope, and magnetometer. The tri-axial accelerometer can measure the rate of change of the body’s velocity through the x-, y-, and z-axis at its local position. It is the most commonly used sensor in HAR [1,4]. We can use the accelerometer data alone to perform HAR, or we can use it in combination with a gyroscope or a magnetometer to improve performance in HAR. It is not typical to develop HAR with only a gyroscope or magnetometer. An essential factor for activity recognition is the sampling rate, defined as the number of readings taken of a piece of data per second, usually expressed in hertz.

Deep learning (DL) is a field of machine learning (ML). Recently, DL has outperformed ML in many applications, such as time series classification, image recognition, speech recognition, object detection, and natural language processing. In object detection, for instance, DL can improve energy efficiency in intelligent buildings. Recognizing the number of people in a room maximizes energy expenditure and guarantees thermal comfort [9]. Traditional ML requires expert human knowledge to extract the relevant attributes from sensor signals and much human effort. For example, in HAR, there is a standard methodology known as Activity Recognition Protocol (ARP), which consists of five steps: preprocessing, segmentation, feature extraction, and classification and evaluation [4,10]. In classic ML, the feature extraction step is done manually by an expert who has studied the essence of the problem and defined the most relevant pieces of information to categorize the problem.

On the other hand, with DL, we can use a neural network (NN) to extract the best features and classify them during its training process, simply giving raw data as input. Convolutional neural networks (CNNs) are a kind of deep architecture. CNNs are so named because of the convolutional layer in their structure, responsible for automatically extracting features during training. In HAR, CNNs can receive both raw data and handcrafted features. Mekruksavanich et al. [11] used CNNs as their base model for biometric user identification (BUI). The model had tri-axial accelerometer and tri-axial gyroscope data as inputs. Their system was composed of two cascade classifiers. The first one was used for activity classification, and the second one performed recognition of the individual.

A significant problem in using DL is the difficulty of interpreting the model. There is a trade-off between performance and simplicity or interpretability. Classic ML models, for example, decision trees, are highly interpretable and explainable but are not performing in complex scenarios. Selvaraju et al. [12] introduced grad-CAM (gradient-weighted class activation mapping), a technique for producing visual explanations for CNN-based models. With grad-CAM, we can better understand model limitations, discerning a “stronger” deep network from a “weaker” network, even when their performance metrics point to similar results. So far, in DL systems, we have sacrificed explanation capability for better performance. However, with grad-CAM, this paradigm has started to change.

HAR plays an essential role in people’s daily lives due to wearables’ popularization. The evolution in these devices’ ability to extract high-level information about the user’s routine is due to the scientific community’s efforts on the topic of HAR. Advances in DL have also played a vital role in this area. DL helps create high-performing models. However, it is not easy even for AI developers to understand how the model makes decisions. When you work with image data, some techniques can assist the designer in understanding the models’ decisions, such as grad-CAM or object recognition models, where the region responsible for the decision making is clearly expressed by bounding boxes. However, this is not extensively explored in HAR and BUI, with time series data from inertial sensors. Most often, the articles only look at performance metrics to estimate the potential and capability of the models. The lack of references using XAI methods in deep learning based on one-dimensional convolutional models applied to HAR and BUI tasks creates difficulties for the AI developer in building a robust model in real-world scenarios.

This paper shows how to generate visual explanations that help to understand the learning of the trained CNN models on both HAR and BUI tasks. We propose a new CNN architecture capable of performing HAR and BUI. We have used the public dataset UniMiB-SHAR [7], which has 17 classes, including daily activities and falls, performed by 30 individuals. To perform the HAR, we trained and compared classifiers with two validation strategies. In the first, known as subject-dependent (SD), we used a hold-out split of 70/30 for training and validation. In the second, known as subject-independent (SI), we used 21 subjects for training and 9 subjects for validation, reaching 0.978 and 0.755 accuracy for each strategy, respectively. We also developed BUI models for each of the 17 activities, differentiating each user who performed the activity, achieving 0.937 average accuracy. Our experiments also revealed a linear correlation with a Pearson coefficient of 0.77 between the results obtained in BUI and the classifier’s performance that executes activity recognition with the SD strategy. In SD, a network learns not only the physical activity but also the signature of the individuals who perform the activities. In addition, we used the grad-CAM technique to produce visual explanations that help understand and explain the convolutive models developed, by examining each architecture to identify possible generalization problems. Finally, we show how the models were affected by the biases of the database. 

Specifically, our contributions are summarized as follows:

(1) We propose a new CNN architecture capable of being used to perform HAR and BUI;
(2) We show a relationship between the network’s performances of HAR with SD and BUI;
(3) We propose a methodology to analyze the learning of one-dimensional CNNs in both HAR and BUI models, using the grad-CAM technique to produce visual explanations that highlight what the model took into account to make a prediction;
(4) We use the visual explanations to identify bias problems in the database.

To the best of our knowledge, this is the first study to present a way to generate visual explanations for one-dimensional convolutional networks applied to HAR and BUI tasks.

The rest of the paper is organized as follows: Section 2 introduces the standard protocol used in developing activity recognition applications. Section 3 presents the related works that perform both HAR and BUI. Section 4 presents the methodology, introducing the dataset, CNN architectures, and the grad-CAM technique. Section 5 presents the results achieved for each experiment performed. Finally, in Section 6, we discuss the results. 

## 2. Activity Recognition Protocol

Most HAR applications apply the standard Activity Recognition Protocol (ARP) [1,4,10,13,14,15]. Basically, ARP consists of five steps: data acquisition, preprocessing, segmentation, feature extraction, and classification and evaluation, as follows.

### 2.1. Acquisition

Responsible for acquiring data from the sensors. At this stage, an application is used to acquire and store the data from the activities, following previously established acquisition protocols. Many works report using a camera or microphone to help label the data [7,14]. The sensor most often used for data capture is the accelerometer since it can measure the direction of movement over time. Many sampling rates have been considered in the literature, but 50 Hz is the most widely used [4].

### 2.2. Preprocessing

Preprocessing can be performed at the time of acquisition or after it. Since the preprocessing method can affect the model performance, executing this step after the acquisition is more common. In preprocessing, data science techniques, digital signal processing (DSP), and machine learning (ML) techniques can improve data quality, identify outliers, and remove noise. With DSP, we can correct sampling problems that occurred during signal acquisition. ML techniques make it possible to analyze the data distribution. With data science, we can treat missing values. 

At this stage, some authors apply filters to correct problems caused by the acquisition process, such as a low-pass filter with a cutoff frequency ranging between 0.1 and 0.5 Hz, in order to isolate and remove the gravitational component because it is a bias that can influence the performance of the model [16].

### 2.3. Segmentation

In this step, the data is processed in smaller segments called windows. To facilitate learning in the model, data quality is further improved. Different activities performed by individuals may have different durations. For example, in the case of the physical activity walking, each individual can take a different period to walk a specific distance. Compared to the walking activity, the jumping activity usually occurs in a shorter time period. With segmentation techniques, we can balance the time windows of the samples, considering each subject’s characteristics and activity. 

In order to make the raw data suitable for use by the model, we start by choosing an optimal window size to recognize all activities. All samples in the dataset should have the same time window. The typical window size is 3 s, used in 56% of recent studies [4]. Next, we have to define the method for windowing the data. The most commonly used techniques are event-defined windowing and sliding windowing. With the former, the windowing process is done around a target event to be detected, such as a spike. The second approach divides the data into fixed-size windows at a constant step. This process may or may not overlap between the samples, meaning that a part of the present sample can include a part of the previous sample. It is common to use a 50% overlap between the samples [4]. 

### 2.4. Feature Extraction

Feature extraction is defined as a process of obtaining information from the signal using mathematical relationships present in it. There are two approaches to this step: handcrafted features and learned features. With the handcrafted approach, we rely on the knowledge of an expert in the area to obtain relevant mathematical relationships capable of differentiating the classes of the problem. In contrast, with the learned features approach, we use machine learning techniques to obtain these features based on data correlations. Although the use of learned features, with raw data input to the networks, has been widely accepted by the scientific community, when working with images, in time series problems such as in HAR, many works are still using handcrafted features [1,4,13]. This is because a deep network requires a higher computational cost than a shallow network. Since ample computational power is unavailable in smartphones and wearables, classical approaches are preferred in most cases, and they do not achieve high performance with raw data.

Handcrafted features can be divided into time, frequency, and symbolic categories. In the time domain, these features are obtained by statistical calculations. Some examples of statistical features are the mean and the standard deviation. Frequency domain features are calculated after applying the Fourier transform to the data. Some examples of characteristics in the frequency domain are the entropy and the sum of the spectral power components. Symbolic features are obtained after a discretization process. Some examples of symbolic features are skewness and kurtosis.

For the learned features approach, some studies are using DL and principal component analysis (PCA) [15,17,18,19,20]. DL algorithms learn features during their training stage. For example, CNNs perform the mathematical convolution operation during their learning process. These nets are trained based on the backpropagation algorithm, which can modify both the kernel coefficients, in the feature extraction layers, and the weights of the classification layer. The kernel coefficients are used to obtain the feature maps. In CNNs, the feature maps are the learned features. Using PCA, which aims to reduce data dimensionality, a set of orthogonal features are extracted from the data, called principal components. These components are the learned features of this approach and can be used as inputs to a classifier. Another way to obtain learned features is with autoencoder networks. The autoencoder is composed of an encoder and a decoder. This neural network architecture seeks to compress the data and reconstruct it from the compressed representation. It is possible to obtain the primary data information, capable of representing it completely, known as latent space. The encoder reduces the dimensionality of the data, while the decoder reconstructs the signal. We can develop autoencoders with CNNs or dense layers. In autoencoders, the latent space is the learned features.

### 2.5. Classification and Evaluation

We have two possible approaches when working on classification systems, model-driven or data-driven [4]. With the model-driven paradigm, we have a solid idea of how the physical system works, and we try to replicate it manually using composition rules that can represent the problem as an equation. With the data-driven paradigm, we can use machine learning to find links and correlations based on many variables and data. The latter is most often adopted in HAR. 

In ML and DL, we use mathematical and statistical techniques to build intelligence capable of solving a problem. Through these techniques, a model does not have to be explicitly programmed. They should be able to learn from the data. Classification is one of the possible applications in this area. Many classifier algorithms use classical ML, such as naive Bayes, support vector machines, decision trees, random forests, and many others. Some classifiers that use DL are the multilayer perceptron, CNNs, residual neural networks, and long-term recurrent networks. The choice of the classification algorithm can dramatically influence classification performance. Generally, classical ML classifiers need the sensor data to be converted into a high-level representation to solve the problem. In other words, these models cannot receive the raw data. The raw input must be converted into representative information to teach a network; this is why feature extraction is essential for classical ML classifiers. They cannot receive raw data because it contains too much unnecessary information and noise. Some classical ML algorithms cannot solve nonlinear problems. When we are developing our classifier, we do not have enough knowledge about whether this will be an “easy” or “hard” task for a neural network. DL systems can solve easy problems and also handle complex problems better. However, they require a large amount of data, while classical ML can handle few data and less complex tasks better. In DL, the complexity of a network is related to the depth of the architecture, i.e., the number of neurons and layers they have.

Once the classification algorithm is chosen, we must choose a validation strategy to divide our dataset into training and testing subsets. This step is needed because ML and DL models must be evaluated with both seen and unseen data, to see if the model has learned to generalize to different instances of the same problem. There are three validation approaches: subject-dependent (SD), subject-independent (SI), and hybrid. The SI strategy does not use end-user data during training. In contrast, the SD and hybrid approaches use this data during the training process. However, the hybrid approach tries to use end-user data in smaller proportions. 

After deciding on the training algorithm and its validation strategy, we must choose a set of metrics that evaluate the developed model’s performance during training and validation. The metric value achieved in the training subset does not represent the model’s performance. We must evaluate the model’s capability based on data not seen in the training, so the metric values achieved in validation are more significant. The most commonly used evaluation metrics in HAR are accuracy, recall, precision, and F1-score, as follows in Table 1.

All metrics are based on true positive (TP), true negative (TN), false positive (FP), and false negative (FN). The TP, TN, FP, and FN concepts are grounded in binary classification. However, they can be extended to a multiclass classification with a one-vs-all strategy. This strategy considers the target class positive, and all other classes are grouped into a negative class. Accuracy is the most widely used metric and represents the overall assertiveness percentage. Accuracy is a simple metric to understand, but when we have a dataset with an imbalance of the classes, it does not indicate the real performance of the classifier. The precision metric shows how the model handles precision in predicting positive samples. Our model recognizes TP samples very well if we have high precision. The recall metric shows how well our model rejects false negatives. This metric is used when there is a high-cost association with an FN. The F1-score measures a balance between precision and recall. This metric is essential when we have an imbalanced class distribution.

## 3. Related Works

This section will discuss related studies in the HAR area, focusing on those that used the UniMiB-SHAR dataset. Next, we discuss the studies that perform BUI based on previous activity recognition, and finally, the grad-CAM method will be presented, together with the studies that are using the technique.

### 3.1. Related Studies in HAR

The first study to implement HAR dates back to the 1990s. Since then, many techniques and frameworks have been proposed. Many sensors can be used in HAR, such as cameras, piezoelectric, GPS, microphone, and IMU.

CNN architectures have been widely used in HAR. They are considered robust due to their scale and transition invariance. Most CNN architectures are encoder-like, i.e., the data loses dimension as it moves into the deeper layers. This contributes to obtaining high-level semantic meanings. These architectures can extract representative features as good as, if not better than, handcrafted methods [15]. They can receive multidimensional data as inputs. For example, a CNN can receive as input a 9-dimensional signal consisting of a tri-axial accelerometer, a tri-axial gyroscope, and a tri-axial magnetometer. They are not limited to working only with raw data. These nets can also receive handcrafted features or learned features from other deep networks. Dong et al. [21] propose HAR-Net, a CNN architecture that can receive handcrafted features on its input. Lee et al. [22] calculate the vector sum of the accelerometer in the x-, y-, and z-axis to obtain a magnitude signal they use as input to a CNN, achieving better performance than a random forest algorithm. Ronao et al. [23] propose a CNN capable of receiving 6-dimensional data as its input, composed of a tri-axial accelerometer and a tri-axial gyroscope. The network achieved 94% accuracy.

Many recent papers have used the UniMiB-SHAR database, as shown in Table 2. All articles used 17 classes and have only raw accelerometer data in the classifier input.

Some papers focus on a new neural network architecture or processing approach, claiming higher performance than their baseline [6,7,24,26]. Usually, these studies show either a benchmark with previous papers that have used UniMiB-SHAR or show the performance of its technique on different datasets. Some articles propose an improvement in the optimization method used to train the deep network or in its internal structure, comparing the performance of their method with traditional approaches [21,25,27].

Mukherjee et al. [24] propose three classification models, named CNN-Net, Encoded-Net, and CNN-LSTM. Each of these models is built using one-dimensional CNNs as a basis. Their input is a two-dimensional matrix obtained from a windowing application with signal overlap. Each neural network performs a vote to decide a class. The class with the highest vote is the predicted class. Micucci et al. [7] used the raw data to input KNN and SVM algorithms. This particular study was the first baseline, as this was the author of the UniMiB-SHAR database. Their study is intended to introduce the dataset and show with a simple implementation that it is possible to perform activity recognition with machine learning algorithms. Tang et al. [25] propose a low computational cost CNN using Lego filters. This work is concerned with the limitation we have when applying deep learning techniques to devices with low computational cost, such as wearable devices. A set of lower-dimensional filters are used in a similar way to Lego bricks. The strategy used showed that it is possible to reduce memory and computation costs using CNN. The authors claim that the model is small, fast, and accurate. LV et al. [26] present a hybrid deep learning network with ConvLSTM architecture. This study joins two deep network architectures, CNN and LSTM networks, to make better use of information extracted by CNN in the temporal domain, also inserting a dense link module to improve the information flow between network layers and a multilayer feature aggregation module to extract features along with spatial domains. The authors also aggregate the features obtained from each convolutional layer according to their importance at different positions in the signal. Teng et al. [21] propose both a new training method and a new architecture for the residual neural network. Residual networks are a specific type of CNN, which generally do not follow a sequential architecture, aggregating information from previous layers in their structure. Their block training uses local loss functions for HAR applications. Instead of common global backpropagation, local cross-entropy loss and local supervised similarity are used to train each residual block independently. The proposed methodology improves classification accuracy and memory requirements during the training phase. Cheng et al. [27] propose improving CNN computation using conditionally parameterized convolution, focusing on real-time HAR using mobile and wearable devices.

### 3.2. Biometric User Identification Using HAR

There are two categories of biometric identification systems. The first is based on static characteristics, including physical features such as the face, fingerprints, and others. The second is based on dynamic features, such as behavioral characteristics, including electrocardiographic signals, voice, or a specific activity. Many studies have explored the possibility of using a smartphone’s built-in sensors to recognize user activity. However, few have explored the potential of this data for BUI.

Mekruksavanich et al. [11] propose identifying the user with different physical activity signals, a tri-axial accelerometer, and a tri-axial gyroscope. Its structure recognizes the user’s activity and then performs authentication. There is one authentication network for each physical activity in the database. The authors used a CNN and an LSTM to achieve 91.77% and 92.43% accuracy, respectively. The datasets used were the UCI human activity recognition dataset and the USD human activity recognition dataset. The first dataset has data from 30 subjects performing six daily activities. The second dataset includes activity data recorded with 14 subjects, 7 males and 7 females, also performing daily activities. The subjects perform 12 daily activities. The window size was 128 samples with 50 Hz sampling frequency in both datasets. 

Juefei-Xu et al. [22] proposed a user identification system based on a previous detection of different walking paces. The walking events are divided into speed categories such as normal and fast. The performance was evaluated in three scenarios. The first, for matching normal versus normal pace, which means that only normal walking samples were considered. The authors achieved a verification rate (VR) of 99.4%, 0.1% for the false acceptance rate (FAR), and 95% for accuracy. In the second scenario, for fast versus fast, they achieved 96.8% for VR, 0.1% for FAR, and 98% for accuracy. In the third, where all samples were used, either normal or fast, the performance dropped to 66.1% for VR, 0.1% for FAR, and 40% for accuracy. FAR measures the average number of false acceptances within a biometric system. This metric is also referred to as the false negative rate. In contrast, VR measures the average number of predicted positive acceptances in the face of ground truth. A tri-axial accelerometer and tri-axial gyroscope are used. The authors used a proprietary database of 36 subjects. Feature extraction by handcrafted methods was employed. The window size used was 1 s. The work showed that the speed at which the user is walking must be considered for correct user identification. 

### 3.3. Gradient-Weighted Class Activation Mapping

Deep networks are usually treated as a black box, where it is impossible to know precisely how the network will behave or why it made a decision. A new branch of research into model explanation called explainable AI (XAI) uses various techniques to explain network decisions. The gradient-weighted class activation mapping (grad-CAM) technique is the most widely used and cited today [28]. 

Sousa et al. [28] used XAI methods for a COVID-19 classifier on CT images. The authors explain that networks can understand undesired artifacts in databases, and model explanation techniques help avoid this problem. They evaluated some standard methods for XAI systems, such as grad-CAM, LIME, RISE, square grid, and direct gradient approaches. The authors show that spurious artifacts may affect some network architectures. The author reveals that high-performance results can lead to a misconception that they do not suffer from bias problems.

Grad-CAM uses a gradient from a target class, flowing into the final convolutional layer of the architecture, to create a coarse location map that highlights the regions of the image that most influenced the model’s decision making [12]. It is known that the deeper convolutional layers can abstract more complex information, so the last convolutional layer is expected to contain the maximum abstraction of the signal. Visualizing this information gives us a clearer idea of what the intelligence has learned [29]. This visualization becomes more interesting when it is possible to focus only on the information that led the network to associate a specific input with a target class with the help of location maps. Grad-CAM emerged as an improvement on the work of Zhou et al. [19], which proposed class activation mapping (CAM) to identify discriminative regions used by restricted classes for image classification with CNN classifiers. However, the CAM technique faces a limitation since it is only applicable for networks that do not have fully connected layers. Grad-CAM is a generalization of CAM, making it possible to apply it to different families of CNNs, including CNNs with fully connected layers, CNNs with structured outputs, CNNs used in tasks with multimodal inputs, and reinforcement learning.

The grad-CAM technique takes a target signal and a class of interest as input. Then, the gradient is calculated for the target class $y^{c}$ with regard to the feature maps of the last convolutional layer $A_{ij}^{k}$. As described in Equation (1), to obtain each neuron’s importance weights $\propto_{k}^{c}$, these obtained gradients are passed through a global average pooling function.

(1) $$\alpha_{k}^{c}=\overset{\mathrm{global}\;\mathrm{average}\;\mathrm{pooling}}{\overset{︷}{\frac{1}{Z}\overset{}{\underset{i}{\sum }}\overset{}{\underset{j}{\sum }}}}\underset{\mathrm{gradients}\;\mathrm{via}\;\mathrm{backprop}}{\underset{︸}{\frac{\partial y^{c}}{\partial A_{ij}^{k}}}}$$

The weights $\propto_{k}^{c}$ represent a partial linearization of the deep network with respect to A, and capture the “importance” of a feature map for a target class. We then compute a weighted combination of the direct activation maps, followed by a ReLU function to eliminate negative contributions. With this, we get the heatmap of the regions of importance in our network $L_{\mathrm{Grad-CAM}}^{c}$, according to Equation (2).

(2) $$L_{\mathrm{Grad-CAM}}^{c}=\mathrm{ReLU}\underset{\mathrm{linear}\;\mathrm{combination}}{\underset{︸}{\left(\underset{k}{\sum }\alpha_{k}^{c}A^{k}\right)}}$$

Grad-CAM is usually applied to images. We will use this technique for a time series of accelerometer data. For this purpose, we have made some modifications to the original method.

We do not need to calculate the global average pooling in two dimensions because it is a time series, only in one dimension, as shown in the following Equation (3):

(3) $$\alpha_{k}^{c}=\overset{\mathrm{global}\;\mathrm{average}\;\mathrm{pooling}}{\overset{︷}{\frac{1}{Z}\underset{i}{\sum }}}\underset{\mathrm{gradients}\;\mathrm{via}\;\mathrm{backprop}}{\underset{︸}{\frac{\partial y^{c}}{\partial A_{ij}^{k}}}}$$

**Figure 1:**
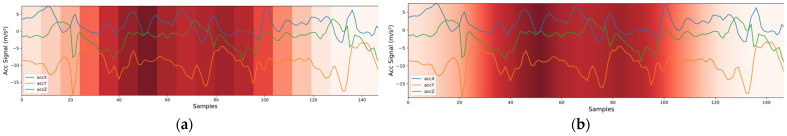
Comparison of nearest-neighbor and bilinear interpolation methods. (**a**) Nearest-neighbor interpolation. (**b**) Bilinear interpolation.

For the heatmap, we do not use the ReLU function. We normalize the signal between 0 and 1. When plotting the heatmap, we use the nearest-neighbor interpolation method to interpolate the signal to the same size as the input signal. Linear interpolation is currently the most widely used with grad-CAM. However, when working with signals, the result is not clear enough when we use this method. As seen in Figure 1, if we are interested in seeing well the regions of importance, the result is more evident when we apply the nearest-neighbor interpolation method because the bands are more evident, while in the bilinear interpolation method, these regions of importance seem to be smoother, making it more difficult to distinguish a very important area from one with intermediate importance.

## 4. Methodology

This section intends to explain the dataset, how the data was trained and divided into each validation strategy, the proposed CNN architectures, and the proposed framework.

### 4.1. Dataset

The UniMiB-SHAR database is composed mainly of data from women aged between 18 and 60 years, with height between 160 cm and 190 cm, and mass between 50 kg and 82 kg. The device used to acquire the data was a Samsung Galaxy Nexus 19250 with Android operating system 5.1.1, using the Bosh BMA220 acceleration sensor. The sampling frequency was 50 Hz, with a time window of 3 s, resulting in 151 samples for each of the 3 axes of the accelerometer. The windowing process was event-based, based on peak detection. Each activity was performed between 2 and 6 times. Two smartphone positions were considered. Half of the participants placed the smartphone in their left pocket, while the other half placed it in their right pocket [7].

The dataset consists of 11,771 data samples. Thirty subjects performed 17 activities, with 9 types of activities of daily living (ADLs) and 8 types of falls. Table 3 shows more information. As shown in the N° Sample column, the dataset is unbalanced. Finally, as shown in the N° subjects column, not all participants performed all activities.

**Figure 2:**
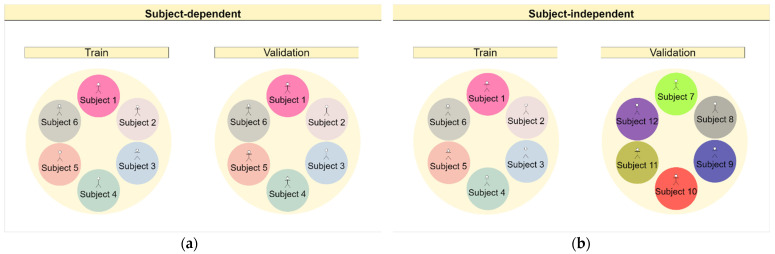
SD validation strategy and SI validation strategy. (**a**) We train and validate with the same subjects represented in the diagram by using circles with the same colors. In our case, we have 70% of the data for training and 30% for validation, which means that, on average, 70% of the total data from each individual were used in training, and the remaining in validation. (**b**) We have different subjects present in the training and validation, represented by the circles in different colors. In our case, we decided that 30% of the total subjects would be used for validation and the rest for training.

Splitting the data into the training and validation subsets for activity recognition depends on the chosen validation strategy. For the subject-dependent (SD) strategy, the data were split with shuffle, using the number 42 as a random state seed, using the Sklearn library in Python. The proportion between training and validation was 70/30. This method can also be known as hold-out. For the subject-independent (SI) validation strategy, the related work usually uses 1 subject in the validation. This method is known as leave-one-subject-out. In our work, considering keeping the same proportion of data used in the subject-dependent strategy, to make the comparison of the two approaches equivalent, we chose to use subjects 1 to 9 in validation and the remaining 21 subjects in training, since 9 represents 30% of the total number of individuals. Figure 2 shows a diagram to illustrate the differences between the chosen validation strategies.

**Figure 3:**
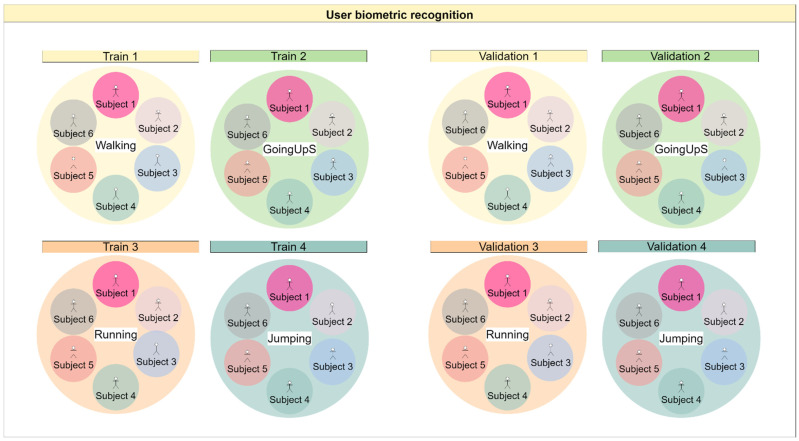
Division of the data for biometric user identification. The larger circles represent separate subsets, and the smaller ones represent different subjects. For example, the running activity is denoted by the orange circle and has a rectangle with the name Train 3 on the left side. There is the same orange circle on the right side, with the name Validation 3, meaning that we have a net trained to recognize individuals who have been trained and validated with just the running activity data.

For the biometric user identification, each physical activity was considered a subset. In the case of the walking subset, all walking samples from all individuals in the dataset were filtered to form the walking subset. Then, with this subset, the same split as in the SD strategy was used, with 70/30 training/validation proportion, using the random state seed 42. Figure 3 clarifies the division performed on the data for training the biometric user identification network.

### 4.2. Architectures

The simulations were performed using 2 convolutional network architectures, CNN1 and CNN2. The architectures were trained and implemented on the TensorFlow 2 framework and had a similar configuration. 

Initially, we will expose what is shared by both architectures. In both, the Adam optimizer with default hyperparameters was used. The models were trained for 300 epochs. Each convolutional block was composed of 2 convolutional layers with 100 feature maps each. The ReLU activation function was used. In each max pooling layer, the pool size and step were 2. The value used in the dropout layer is 0.5. The softmax layer has *n* neurons, representing the number of classes for the particular problem. We used a custom callback to make checkpoints after each training epoch to obtain the best model based on the macro F1-score metric on the validation set. The batch size was 256 samples. Both architectures had received the same signal at their inputs with the shape of 151 × 3.

**Figure 4:**
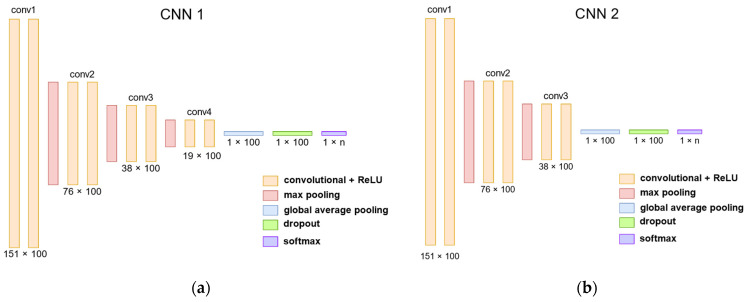
The CNN architectures implemented with TensorFlow 2. (**a**) CNN1 architecture with 4 convolutional blocks. (**b**) CNN2 architecture with 3 convolutional blocks.

Next, we point out the differences between the two architectures. The first architecture has 4 convolutional blocks consisting of 2 convolutional layers, while the second has 3 convolutional blocks. In CNN1, the kernel size was 8, while in CNN2, it was 4. Figure 4 shows both architectures.

### 4.3. Proposed Framework

**Figure 5:**
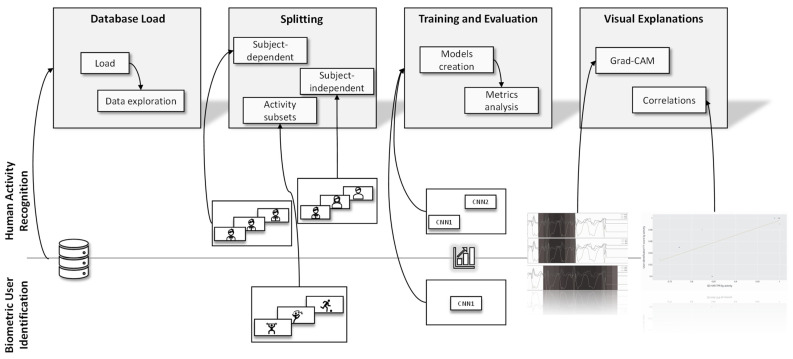
Summary of the framework used for the work. In the top part, we have the processes applied in HAR, in the bottom part, the processes applied in BUI.

To summarize, we propose the framework illustrated in Figure 5. In the database loading step, we performed the database processing and an exploratory analysis of the data. This exploratory analysis allowed us, for example, to identify that not all individuals performed all activities in the UniMiB database. After this, the data was subdivided in the splitting step according to the appropriate validation strategy. In HAR, we had the SD and SI strategies, while in BUI, we had the generation of a subset with physical activity. Then, the models were implemented in the training and evaluation step, and their results were contrasted using performance metrics. We used the CNN1 and CNN2 architectures for HAR, and for BUI, only the CNN1 architecture. 

The framework is similar to the Activity Recognition Protocol, adding the step of visual explanation. This step allowed a more comprehensive notion of the limitations of the models and database. We generated two graphs, the first with grad-CAM and the second with a correlation between HAR SD and BUI. Using grad-CAM, visual explanations were generated for each class in the dataset, which allowed biases in the database to be identified. Furthermore, these visual explanations gave a clearer idea of the model’s potential. 

## 5. Results

In this section, the model results for HAR and BUI will be briefly presented and discussed.

### 5.1. HAR

We evaluated and trained the CNN1 and CNN2 architectures with the SD and SI strategies. We considered macro average and weighted average metrics to better compare our results with other works. The difference between them is that the former only considers the impact achieved by the metric in each class. In contrast, the latter considers the result of the metric in each class weighted by the number of samples evaluated. The first deals with an unbalanced system better since it does not distinguish between classes with more extensive data. In contrast, the second one provides a more general system idea, as long as the distribution of the classes in the dataset is equal to the distribution of this data in the real world.

Table 4 and Table 5 show the performance of each network in more detail, considering the SD and SI strategies, respectively. Appendix A shows the detailed performance of the CNN1 network for the two validation strategies.

The results for the SD validation strategy were significantly higher than those achieved with the SI strategy for both trained architectures, considering all metrics. This result was already expected since previous studies point out that the SI validation method is more challenging and is closer to the actual result that a network will have since the network will not have prior knowledge about how individuals perform the activity [4].

In the two scenarios evaluated, CNN1 was superior to CNN2. However, with the SD strategy, both achieved similar results. The difference between the values of macro F1-score obtained by the two architectures differed by only 0.2%. In the SI strategy, it is already possible to see a more significant difference in performance of approximately 2% for the macro F1-score metric and 4% for the accuracy metric.

Although the focus of our article is not to get the best possible architecture in the areas of HAR and BUI, the results achieved are as good as, or even better than, the articles presented in Table 2. Although the validation methods and subsets are different, the model’s excellent performance is worth noting. Observing the accuracy metric, our work achieved the same accuracy as the best work with the SD strategy, 0.978, using the CNN1 network. The difference is that this result was achieved with cross-validation. Considering that cross-validation overestimates network performance, the result obtained with the hold-out method is significant [10]. A complete analysis of previous works can be done by looking at Table 2, so we included all our performance metrics obtained with CNN1.

The result for the SI strategy was 0.755 accuracy, which is also close to the baseline. Again, the results achieved in this article are more significant, since the best result was achieved with the LOSO method, which has only one individual in the validation. Ours has nine individuals in the validation set.

The performance metrics show that the best validation strategy was SD, and the best network was CNN1. In Section 6, we will use other evaluation and validation methods to contrast these architectures and choose both the best model and the best validation approach.

**Figure 6:**
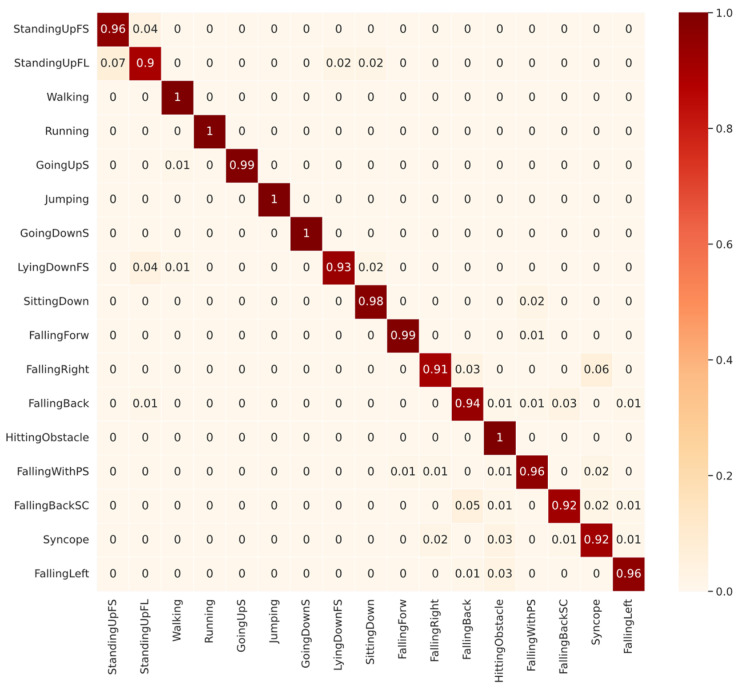
Confusion Matrix of CNN1 with subject-dependent 70/30 for all 17 classes of UniMiB-SHAR dataset.

Next, we will analyze the diagonals of the confusion matrices shown in Figure 5 and Figure 6. The diagonal is our recall metric, previously defined in Table 1. This metric is also called the true positive rate (TPR). As shown in Figure 6, for CNN1 validated with SD, among the ADLs, the lowest-performing class was StandingUpFL with 90% TPR, followed by LyingDownFS with 93% TPR. The confusion about StandingUpFL was quite reasonable, since 7% of the wrongly predicted samples were mistaken for StandingUpFS, which are indeed similar ADLs. However, among the LyingDownFS confusion, there does not seem to be a logical sense, since most of the confusion, approximately 4%, occurred with the StandingUpFS class. Observing the falling performance of classes, confusion seems to occur between the different types of falling. The lowest-performing class was the FallingRight class with 91% TPR, most frequently confused with the Syncope class.

**Figure 7:**
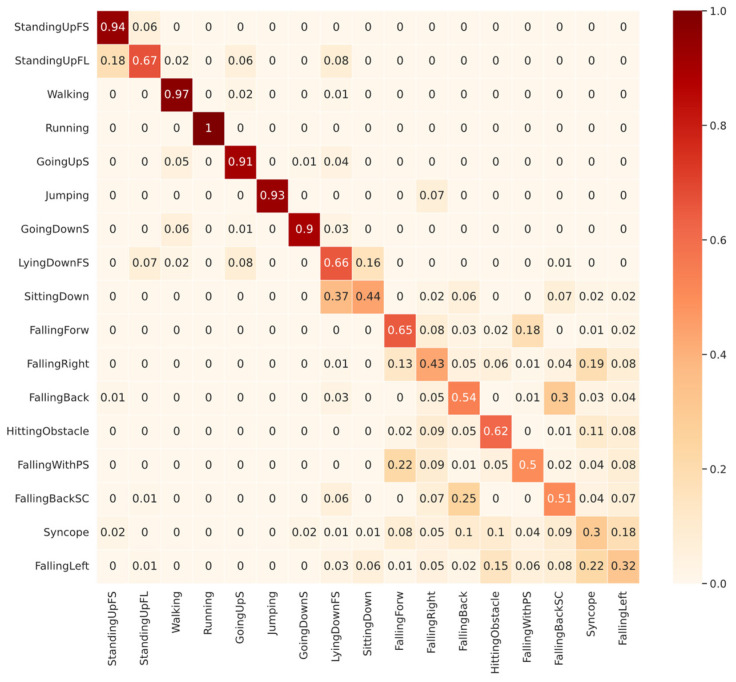
Confusion Matrix of CNN1 with subject-independent with 9 subjects in validation for all 17 classes in UniMiB-SHAR dataset.

Figure 7 shows that among the ADLs, the lowest performance for the CNN1 validated with SI came from SittingDown with 44% TPR, followed by LyingDownFS with 66% TPR. The SittingDown confusion shows a possible problem in network learning, where 37% of the samples were confused with GoingDownS, which are entirely different activities. Most of the confusion in the LyingDownFS class, representing 16% of the total, was with the SittingDown class, strengthening the idea that the features learned from the SittingDown class were inappropriate. In Section 6, we will show that the SittingDown and LyingDownFS classes have some bias problems that can affect the network’s learning. Among the falling classes, the same pattern identified in SD was found. Most of the confusion occurs among the falling types, but also with ADL classes, as in FallingBackSC, in which 6% of the samples were confused with LyingDownFS.

In general, in SI, for some classes, the features learned by some subjects were enough to classify the activity performed by the other subjects, such as the Running class. In SD, as the network sees samples from all the people, the classification becomes easier, reflecting higher performances. However, it is not possible to know if these features were relevant enough to perform well for subjects outside the database since we do not have an independent portion of the data with the same distribution to validate the results.

### 5.2. Biometric User Identification

**Figure 8:**
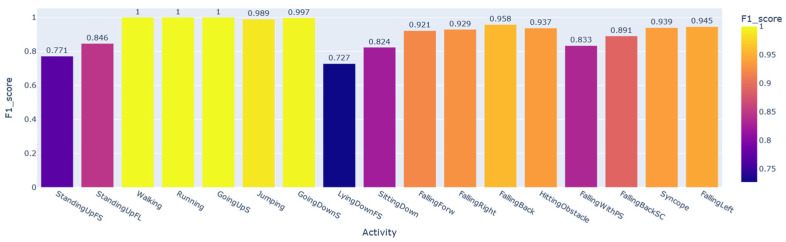
General BUI performance for each activity considering the macro F1-score.

**Figure 9:**
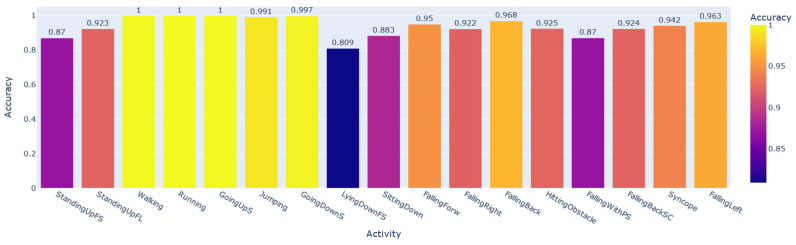
General BUI performance for each activity considering the accuracy.

To prove that the HAR data samples carry a large portion of the user’s personal information, we trained and evaluated the CNN1 architecture to perform the subject identification according to the activity developed by the individual. This system in production should be accompanied by a prior classifier that detects the activity performed by the user and only then recognizes the subject, since each identification AI obtained was trained and validated by filtering the database by activity. Using the SD data split shown in Figure 2, we modified only the number of neurons in the last layer of CNN1, softmax, for the number of subjects that performed each activity, according to the column N° subjects in Table 3. Seventeen networks were obtained, one for each activity. Figure 8 shows the performance of each AI developed based on the macro F1-score metric. In contrast, Figure 9 shows the same performance based on the accuracy metric.

In some activities, the user’s personal information is more significant than in others. For example, in Walking samples, it is possible to recognize each of the individuals with a 100% macro F1-score. At the same time, for the LyingDownFS subset, this value drops to 73%. Among the most common everyday physical activities that achieved the highest performances were Walking, Running, GoingUpS, and GoingDownS.

Later in the discussion section, we will associate the performance in recognizing the activity with the performance in identifying the subject.

Overall, we can see that it is possible to recognize the individuals well, with more than 91% average of all macro F1-scores obtained for each subset. Considering the accuracy, this percentage rises to 93%. In the next section, we will demonstrate that the StandingUpFS, StandingUpFL, LyingdownFS, and SittingDown classes are activities that suffer from database bias problems. These were precisely the lowest-performing subsets of our AI for user identification, showing that the performance of this network for identification would be even higher if we disregarded the performance for these activities.

## 6. Discussion

In this section, we explain the results obtained by each trained network, either for HAR or BUI. We explain possible reasons for the difference in performance between the SI and SD strategies using analyses involving the identification networks. We then use the visual explanations of the grad-CAM technique to visualize what the networks took into account for decision making. Finally, with the same technique, we find inconsistencies in our database and show how these inconsistencies affected learning in the networks.

### 6.1. Explaining the HAR and BUI Correlationship

Table 3 shows that not all subjects performed all activities in the UniMiB-SHAR dataset. Furthermore, in Table 2, many papers used the SI approach with a leave-one-subject-out validation strategy. This validation approach is not the appropriate strategy for this scenario, since most machine learning models need independent and identically distributed data (IID) [30]. Since not all individuals performed all activities in this dataset, using this strategy does not meet the criteria of having IID data.

Furthermore, many papers claim to have surpassed the performance baseline and to be the current state of the art of the UniMiB-SHAR dataset [20,24]. However, some works do not use the same validation strategy as their baseline, making the results noncomparable. Some papers use the same validation strategy, but with different subsets of data, by not using an equivalent data split [21,25]. For example, most data splits are done randomly, but every split must be based on the same random seed to ensure the reproducibility of the results. However, some authors do not pay attention to this fact, obtaining no equivalent subsets, which implies that, in some cases, we may have similar samples in the training and validation sets, making the performance high. In contrast, there may be fewer similar samples in the training and validation subsets in other cases, raising the problem of generalization of the network, probably resulting in lower performance.

**Figure 10:**
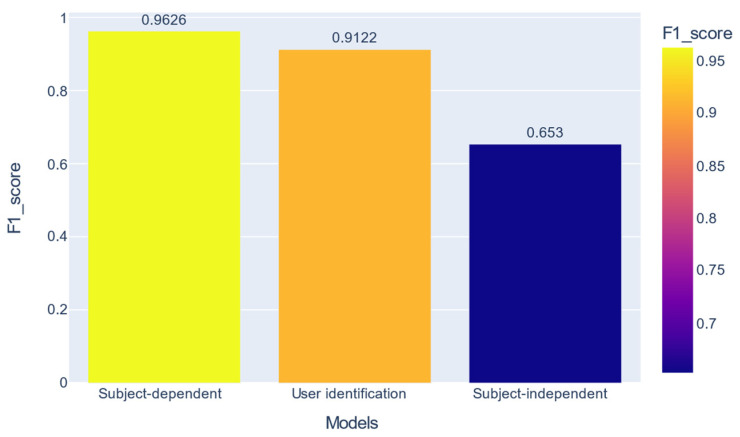
Comparison between the best performances obtained with HAR with SD, biometric user identification, and HAR with SI.

In the previous section, it was shown that the nets recognize all 17 activities well, but there is a difference of over 31% between the two validation strategies for the macro F1-score metric. We can also see a high performance in user identification per activity. Figure 10 shows a combination of these performances.

Previous work has mentioned that all activity data contain personal user information [31]. Our work shows that the user signature is more potent in some activities than others. For example, in the Walking activity, we can identify the user with 100% F1-score macro, while in the StandingUpFS activity, this metric drops to 77.1%.

**Figure 11:**
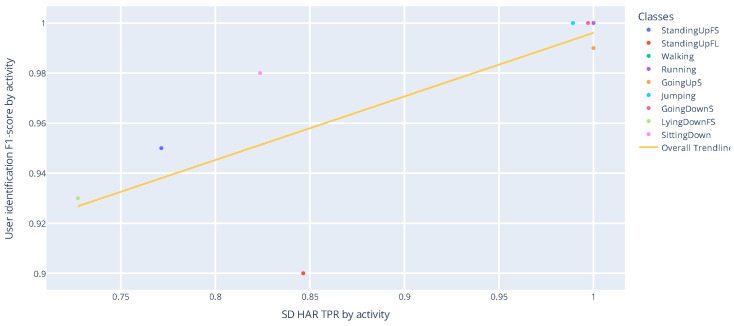
Correlation between the performance of user identification and SD HAR for ADLs.

Figure 11 shows a correlation between the recognition performance of ADLs with the SD strategy, as well as the user identification performance for these ADLs. The Pearson coefficient and the *p*-value for testing noncorrelation are 0.775 and 0.014, respectively. Pearson’s coefficient measures the linear relationship between two distributions. Correlations of −1 and +1 imply a perfect positive correlation and a perfect negative correlation. The *p*-value exposes the probability of these correlations occurring at random.

The strong correlation obtained for ADLs may explain the difference between the SD and SI validation strategies. Basically, with the SD strategy, the network learns the activities’ patterns and personal signatures from the subjects. The result is that the network may memorize how all individuals do the activities and does not learn the correct patterns to recognize the activity.

**Figure 12:**
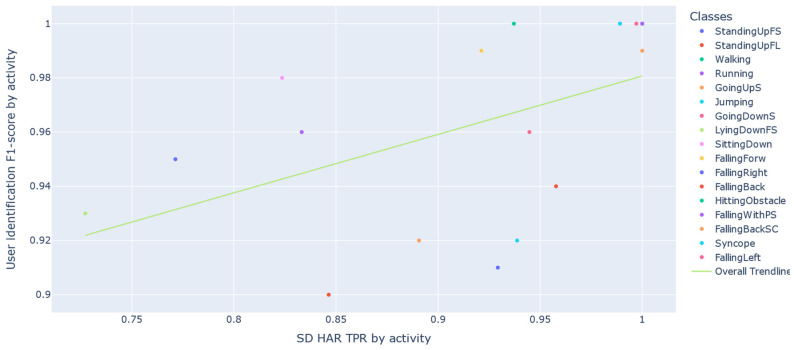
Correlation between the performance of user identification and SD HAR.

As shown in Figure 12, considering ADLs and Falls, the Pearson correlation and *p*-value obtained were 0.504 and 0.039. When we consider falls, the correlation is weaker. However, falls are not commonly used to recognize the user in the same way as ADLs.

In general, in each activity analyzed, the performance of the classifier trained with SD may not indicate how well this classifier will recognize the activities in real-life scenarios.

### 6.2. Interpreting CNN HAR Models with Gradient Class Activation Mapping

**Figure 13:**
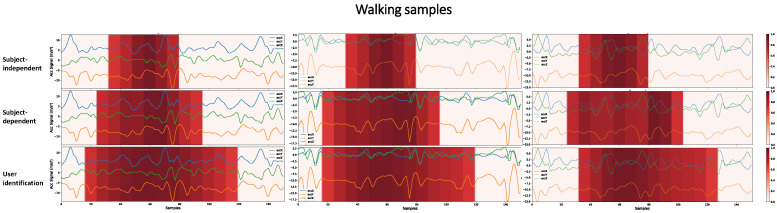
Higher-importance segments for decision making in the CNN1 architecture, for HAR with SI, HAR with SD, and user identification, considering Walking samples with different subjects. The darker the area, the more critical it is for decision making. To obtain these heatmaps, a threshold of 0.7 was considered in the grad-CAM result in order to make more evident the regions of greater importance.

Interpreting the models’ predictions is essential to evaluate their generalization capability and understand their limitations. Grad-CAM provides a way to perform this analysis based on visual information about the regions of the input signal that most influence the model’s decision making. Figure 13 shows what the CNN1 architecture has learned and considered to predict Walking activity.

There are key patterns that help identify each activity. A network can learn this key event or other hidden patterns to identify an activity. However, the larger the region that the architecture considers for decision making, the less likely it is to have learned only these key patterns. We are aiming to demonstrate that when the network, in decision making, considers a vast region of the signal, there may be a portion of the individuals’ personal information that was learned during training. A user identification network must consider a larger part of the signal, since it must learn the person’s digital signature in addition to the key events of the activities. Figure 13 shows that the SI, SD, and user identification networks gradually consider larger portions of the signal to make predictions. It is probable that the more extensive the region is taken into account in decision making, the more personal information from the user the network is learning.

**Figure 14:**
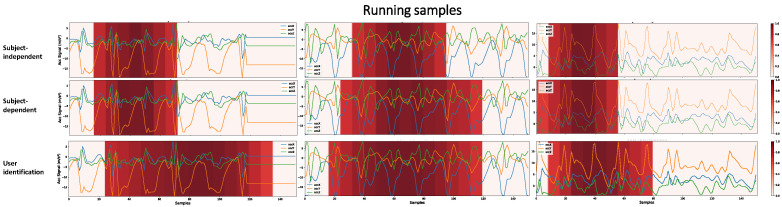
The most critical decision-making segments in the CNN1 architecture for HAR with SI, HAR with SD, and user identification, considering samples of the Running class with different subjects. To obtain the heatmaps, a threshold of 0.7 was considered in the grad-CAM result in order to make the most important regions more evident.

Some activities have multiple key events in a single sample. For example, in the Running activity, where key events are associated with steps, in the 3 s period of a sample, the number of key events is greater than in the Walking activity. Since CNN1 has only 19 × 1 resolution in its last layer, if our class has many key events, the network for decision making considers more extensive regions of the signal, as shown in Figure 14. Thus, for this activity, the HAR networks with SI and HAR with SD obtained closer results than in Figure 13, not necessarily because the network learned the user’s characteristics. In this case, the network learned an activity with multiple key events. Furthermore, it is still possible to see that the HAR with SI networks, for decision making, generally takes into account a less extensive region of the signal. At the same time, the authentication network continued to show the same pattern as the previous activity, considering a large region of the signal as important.

**Figure 15:**
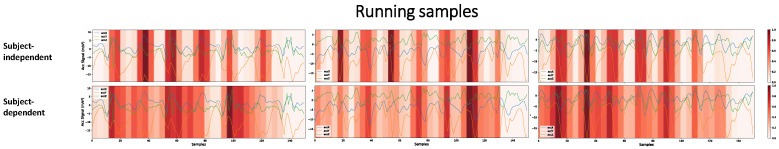
Higher-importance segments for decision making in the CNN2 architecture, for HAR with SI and HAR with SD, considering samples of the Running class with different subjects. To obtain the heatmaps, no threshold was considered in the grad-CAM result in order to make more transparent the smaller, but significant, importance zones that the SD strategy takes into account.

As shown in Figure 3, the CNN2 architecture, if compared to the CNN1 architecture, has a higher resolution (number of neurons) in the last convolutional layer. While CNN1 has 19 × 1, CNN2 has 38 × 1 resolution. Figure 15 shows that this higher resolution allows the network to learn key events more precisely. We contrast the learning between the SI and SD approaches through this figure. We remove the visualizations from the user identification networks since it always considers a large signal region, as shown in Figure 13 and Figure 14. We can see that the SI and SD strategies learn key events, but the SD network also gives secondary importance to the signal as a whole. The user’s signature is what the network is learning among the key events. The influence this may have on the final result is that the network may memorize how users in the database perform activities rather than learning the specific physical activity patterns. The portions of secondary importance between the key events in the SI network are less spread out than in the SD network. 

**Figure 16:**
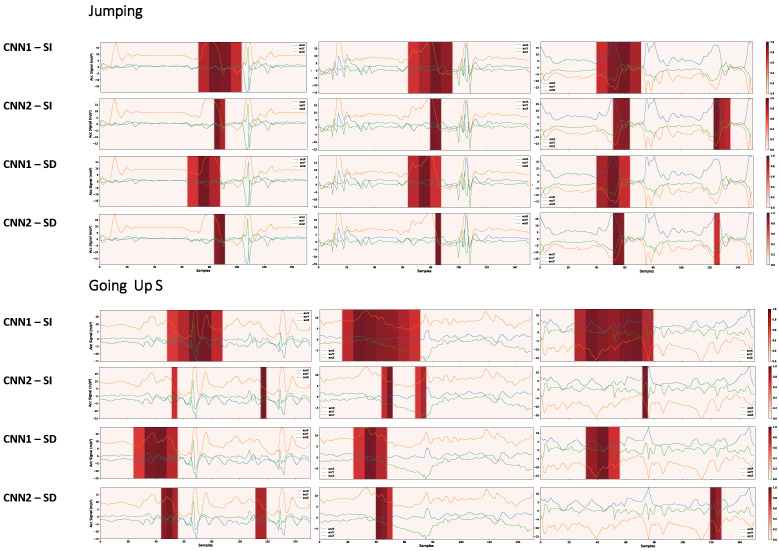
Most important portions for decision making in the CNN1 and CNN2 networks with the SD and SI strategies, for samples of the Jumping and GoingUpS activities. To obtain the heatmaps, a threshold of 0.7 was considered in the grad-CAM result to make the critical regions more evident.

Next, as shown in Figure 16, the learning of the CNN1 and CNN2 architectures is compared using the two validation strategies.

It can be seen that, often, the CNN2 network for decision making takes into account a smaller signal region. Having a smaller region does not always mean we have a better model. For example, the CNN1 network learned more complex features about the signal when, for decision making, it took into account a more extensive region of the signal. At the same time, the CNN2 network learned fewer complex events that can more easily occur in other samples outside the database, leading to incorrect predictions.

A model may be very robust when evaluated through its performance metrics. However, it may not be able to generalize well in real-world scenarios. As shown in Figure 16, although the architectures CNN1 and CNN2 trained with the SD strategy learn different features, their performance metrics differed by only 0.2%. The CNN1 network can learn more complex features than the CNN2 network. We also conclude that the CNN1 network appears more robust in that it does not give importance to key events as short as CNN2, making this network less susceptible to noise. This analysis would not be possible without the grad-CAM technique’s visual exploration.

### 6.3. Identifying Dataset Bias Problems

A machine learning model can learn from data and is, therefore, called data-driven. However, if the data has some problem or some bias, the model inevitably learns this noisy information.

Analyzing the dataset is an important task, but it becomes challenging with extensive databases. Some problems are easy to identify, such as missing data. However, there can also be more complex situations that are difficult to map. Visual exploration through grad-CAM can also find bias problems in the database.

Selvaraju et al. [12] used these techniques to find a problem in their database. The authors were developing an AI model to recognize doctors and nurses, regardless of the gender of the person. The model took an image as input and made a binary classification between the two possible classes. Evaluating the model on its dataset, the performance metrics showed that the model generalized well. However, the result was quite different when testing images outside the dataset. So, they decided to apply grad-CAM to help solve the problem. Analyzing the regions of importance for predicting the images, the authors observed that the model looked at the person’s face and hairstyle to distinguish between doctors and nurses. This explained why the model classified most men as doctors and most women as nurses. The model learned a gender stereotype in its database: mostly male doctors and mostly female nurses. After this analysis, the authors added more images of female doctors and male nurses, solving the problem.

**Figure 17:**
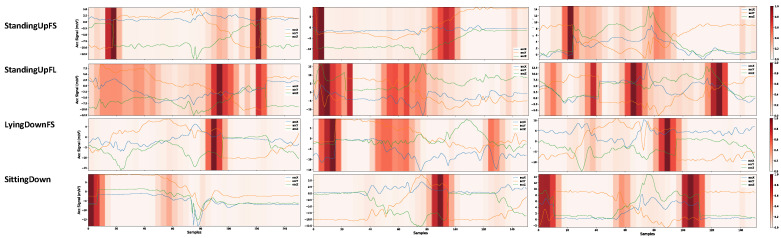
Possible bias problems in the dataset were identified from visual analysis of the grad-CAM in the samples. No threshold was considered for the heatmaps. We can see that the network gave too much importance to discontinuities when predicting. This will probably reflect a problem in the generalization of the model.

In this work, we used the grad-CAM visual explanations to identify possible bias issues in the UniMiB-SHAR database. Analyzing sample predictions from the CNN2 architecture, we found some strange events in the StandingUpFS, StandingUpFL, LyingDownFS, and SittingDown classes, as shown in Figure 17. This same pattern was repeated in both the SD and SI approaches for the CNN2 architecture. However, since the SD approach achieved higher performance metrics, we chose it to evaluate the results.

Taking the SittingDown class as an example, the network concentrated its prediction on noisy segments. For the first sample of this class, we have an abrupt discontinuity on the y-axis and z-axis. For example, in Figure 17, for the first StandingUpFS sample, in the upper left corner between segments 18 and 19, the signal rises from −10 to 1.3 on the y-axis and falls from 2.8 to −9.2 on the z-axis. Although this sample is from the sitting activity, the user cannot make such a rapid movement. Considering the sampling frequency of 50 Hz, the user would have to perform this movement in 0.02 s. These sections of discontinuities appear in some samples of the three classes mentioned. The discontinuities can occur for several reasons. It can be a problem in the sensor, a problem in the application collecting the data, or even a problem in the windowing process for these samples. Sensor problems can be due to poor calibration. Problems in the collection application may cause the sampling frequency to not be constant, or there may be time lapses due to a crash in the collection application.

Regarding the windowing problems, this may have occurred when assembling the dataset, where a section of a sample may have been merged with the subsequent sample. For example, for each activity, six trials were performed. The windowing process may have caused samples from one trial to be merged with segments from the other trial. Based on our observation, these discontinuities may occur in all three axes or only in the y- and z-axis.

We noticed that the model did not learn the important events to detect the activity, it only focused on the parts where these noises occur. Our network has learned to look at discontinuities to differentiate samples from these four classes. In a real scenario, the network will not find such a pattern, and the result tends to be bad.

In general, both architectures were influenced by the discontinuities present in the dataset. As expected, the CNN2 network was more affected as it generally focuses on smaller signal regions.

Discontinuity problems were also found in the fall classes, affecting the network performance. Analyzing the SD and SI confusion matrices presented in Figure 6 and Figure 7, it was observed that the classes with the highest performance difference had a bias problem in the dataset. Among the ADLs, the classes with the most significant performance differences were SittingDown, LyingDownFS, and StandingUpFL, with 54%, 27%, and 23% differences in SD and SI performance, respectively, considering the TPR metric. Bias problems were identified in the three mentioned classes, as shown in Figure 17. The performance in the fall classes differed among all classes, but the same issues of discontinuities were identified. Thus, we can conclude that if there is a high difference, in a target class, between the performances of HAR networks in SI and SD, the dataset probably suffers from a bias problem in that class.

## Figures and Tables

**Table 1:** Most commonly used metrics in HAR systems.

Metric	Equation
Accuracy	$\frac{\mathrm{TP}+\mathrm{TN}}{\mathrm{TP}+\mathrm{TN}+\mathrm{FP}+\mathrm{FN}}$
Recall	$\frac{\mathrm{TP}}{\mathrm{TP}+\mathrm{FN}}$
Precision	$\frac{\mathrm{TP}}{\mathrm{TP}+\mathrm{FP}}$
F1-score	$2\times \frac{\mathrm{Precision}\times \mathrm{Recall}}{\mathrm{Precision}+\mathrm{Recall}}$

**Table 2:** State-of-art studies using the UniMiB-SHAR dataset; N × M—N is the input size and M is the input dimension; SD—subject-dependent; SI—subject-independent; CV—cross-validation; LOSO—Leave-one-subject-out.

Author	Model	Input (N × M)	Validation Strategy	Best Performance
Micucci et al. [7]	KNN and SVM	51 × 3 and 151 × 3 samples	SD: CV-5 and SI: LOSO	SD with 151 × 3—0.830 of MAA * with KNN SI with 151 × 3—0.547 of MAA with KNN
Mukherjee et al. [24]	Ensemble of CNN models	151 × 3 samples	SD: CV-5	SD: 0.926 of accuracy
Tang et al. [25]	CNN	151 × 3 samples	SD: Hold-out 70/30	SD: 0.775 of weighted F1-score
Serrão et al. [6]	GRU and CNN-2D	151 × 3 samples	SD: CV-5 and SI: LOSO	SD: 0.955 of MAA and 0.978 of accuracy with GRU SI: 0.714 of MAA and 0.798 of accuracy with CNN-2D
LV et al. [26]	ConvLSTM	151 × 3 samples	SD: CV-5 and SI: LOSO	SD: 0.953 of MAA and 0.973 of weighted F1-score SI: 0.784 of weighted F1-score
Teng et al. [21]	Resnet (CNN)	151 × 3	SD: Hold-out 70/30	SD: 0.806 of weighted F1-score and 0.809 of accuracy
Cheng et al. [27]	CNN	151 × 3 samples and 151 × 1 (magnitude)	SD: Hold-out 70/30	SD: 151 × 3: 0.886 of accuracy and SD: 151 × 1: 0.7731 of accuracy

* MAA = Mean Average Accuracy.

**Table 3:** UniMiB-SHAR dataset details.

Action	Label	Activity	N° Samples	N° Subjects
	StandingUpFS	Standing up from Sitting	153	24
	StandingUpFL	Standing up from Laying	216	28
	Walking	Walking	1738	29
	Running	Running	1985	30
	GoingUpS	Going upstairs	921	30
ADL	Jumping	Jumping	746	30
	GoingDownS	Going downstairs	1324	30
	LyingDownFS	Lying down from sitting	296	29
	SittingDown	Sitting Down	200	28
	FallingForw	Falling forward	529	30
	Falling Right	Falling rightward	511	30
	Falling Back	Falling backward	526	30
Falls	Hitting Obstacle	Hitting obstacle	661	30
	Falling With PS	Falling with protection strategies	484	30
	Falling Back SC	Falling backward-sitting-chair	434	30
	Syncope	Syncope	513	30
	Falling Left	Falling leftward	534	30

**Table 4:** Performance of CNN1 and CNN2 in HAR, with the SD strategy and 70/30 split.

Model	Macro Average			Weighted Average			Accuracy
	Precision	Recall	F1-Score	Precision	Recall	F1-Score	
CNN1	0.9634	0.9624	0.9626	0.9781	0.9779	0.9779	0.9779
CNN2	0.9612	0.9616	0.9610	0.9771	0.9768	0.9768	0.9768

**Table 5:** Performance of CNN1 and CNN2 in HAR, with the SI strategy and 9 subjects on validation.

Model	Macro Average			Weighted Average			Accuracy
	Precision	Recall	F1-Score	Precision	Recall	F1-Score	
CNN1	0.6513	0.6631	0.6530	0.7619	0.7553	0.7565	0.7553
CNN2	0.6361	0.6361	0.6328	0.7321	0.7177	0.7207	0.7177

## Data Availability

The UniMiB-SHAR dataset can be found on http://www.sal.disco.unimib.it/technologies/unimib-shar/ (accessed on 23 April 2022).

## Appendix A

**Table A1:** CNN1—Subject-dependent detailed performance macro average.

Class	Precision	Recall	F1-Score	Support
StandingUpFS	0.9216	0.9592	0.9400	49
StandingUpFL	0.9000	0.9000	0.9000	60
Walking	0.9943	1	0.9971	523
Running	1	0.9984	0.9992	614
GoingUpS	1	0.9928	0.9964	276
Jumping	1	1	1	228
GoingDownS	0.9975	1	0.9987	393
LyingDownFS	0.9875	0.9294	0.9576	85
SittingDown	0.9516	0.9233	0.9672	60
FallingForw	0.9935	0.9935	0.9935	155
FallingRight	0.9653	0.9085	0.9360	153
FallingBack	0.9119	0.9416	0.9265	154
HittingObstacle	0.9384	1	0.9682	198
FallingWithPS	0.9762	0.9609	0.9685	128
FallingBackSC	0.9449	0.9160	0.9302	131
Syncope	0.9193	0.9193	0.9391	161
FallingLeft	0.9753	0.9573	0.9662	164

**Table A2:** CNN2—Subject-dependent detailed performance macro average.

Class	Precision	Recall	F1-Score	Support
StandingUpFS	0.9787	0.9388	0.9583	49
StandingUpFL	0.9219	0.9833	0.9516	60
Walking	1	1	1	523
Running	1	0.9984	0.9992	614
GoingUpS	1	0.9964	0.9982	276
Jumping	1	1	1	228
GoingDownS	0.9949	1	0.9975	393
LyingDownFS	0.9868	0.8824	0.9317	85
SittingDown	0.9077	0.9833	0.9440	60
FallingForw	0.9730	0.9290	0.9505	155
FallingRight	0.9097	0.9216	0.9156	153
FallingBack	0.9536	0.9351	0.9443	154
HittingObstacle	1	0.9949	0.9975	198
FallingWithPS	0.9323	0.9688	0.9502	128
FallingBackSC	0.9084	0.9084	0.9084	131
Syncope	0.9375	0.9317	0.9346	161
FallingLeft	0.9357	0.9756	0.9552	164
